# Diffuse Large B-Cell Lymphoma Presenting as an Infiltrative Masticator Space Lesion Mimicking Inflammatory Disease: A Case Report

**DOI:** 10.7759/cureus.115313

**Published:** 2026-08-27

**Authors:** Ana Barreira Cabrita, Irma Melendez

**Affiliations:** 1 Family Medicine, Unidade de Saúde Familiar (USF) Santiago de Palmela, Setúbal, PRT; 2 Family Medicine, Unidade de Saúde Familiar (USF) Castelo, Unidade Local de Saúde da Arrábida, Setúbal, PRT

**Keywords:** differential diagnosis, diffuse large b-cell lymphoma, dlbcl, extranodal lymphoma, head and neck lymphoma, inflammatory process, mandibular mass, masticator space, non-hodgkin lymphoma, r-chop

## Abstract

Diffuse large B-cell lymphoma (DLBCL) is the most common subtype of non-Hodgkin lymphoma in adults. Although extranodal involvement is frequent, presentation as an infiltrative lesion of the masticator space is rare and may mimic inflammatory, odontogenic, salivary gland, or soft tissue disorders, leading to diagnostic delay.

We report the case of a 34-year-old woman who presented with progressive right mandibular swelling, facial paresthesia, trismus, weight loss, and night sweats. Initial computed tomography suggested an inflammatory process, whereas magnetic resonance imaging revealed an infiltrative lesion involving the right masticator space, deep lobe of the parotid gland, and ipsilateral cervical lymph nodes. Fine-needle aspiration and an initial biopsy were inconclusive, requiring excisional lymph node biopsy for definitive diagnosis. Histopathological examination confirmed germinal center B-cell-type DLBCL associated with high-grade follicular lymphoma and CD30 expression. The patient was classified as having limited-stage disease and received four cycles of rituximab, cyclophosphamide, doxorubicin, vincristine, and prednisone (R-CHOP) chemotherapy, achieving complete metabolic remission. More than three years after treatment completion, she remains disease-free.

This case highlights the importance of including lymphoma in the differential diagnosis of infiltrative lesions of the masticator space and emphasizes the need for adequate tissue sampling when initial investigations are inconclusive.

## Introduction

Diffuse large B-cell lymphoma (DLBCL) is the most common aggressive subtype of non-Hodgkin lymphoma, representing approximately 30% of all lymphoma cases worldwide [1]. Despite a common morphology characterised by the diffuse proliferation of mature large B cells, these tumours are clinically and biologically very heterogeneous. Most DLBCLs originate in lymph nodes, but ≤40% initially present in extranodal sites [2].

Head and neck manifestations are relatively common among extranodal lymphomas; however, primary involvement of the masticator space is distinctly uncommon [3,4]. The masticator space contains the muscles of mastication, mandibular ramus, mandibular division of the trigeminal nerve, and associated vascular structures [4]. Lesions arising in this compartment often present with facial swelling, pain, trismus, or sensory disturbances and may mimic inflammatory, odontogenic, salivary gland, or soft tissue neoplastic conditions [5].

Because imaging findings are frequently nonspecific, diagnosis may be delayed or require multiple biopsies before adequate tissue is obtained for histopathological evaluation [4]. Early recognition is essential because DLBCL is potentially curable with immunochemotherapy, particularly when diagnosed at a limited stage [1,2]. We present a case of DLBCL involving the masticator space in a young woman whose diagnosis required repeated tissue sampling before definitive histological confirmation.

## Case presentation

A 34-year-old woman with a history of high-grade cervical intraepithelial neoplasia treated with cervical conization several years before presentation and euthyroid thyroiditis presented with progressive swelling of the right mandibular region.

Symptoms began approximately five months before the initial presentation with gradual enlargement of the right mandibular area, subsequently extending to the ipsilateral submandibular and lateral cervical regions. Associated manifestations included facial paresthesia, pain on palpation, progressive limitation of mouth opening, anorexia, fatigue, approximately 10 kg of unintentional weight loss, and night sweats. No dysphagia or fever was reported.

An initial computed tomography (CT) scan performed at the initial diagnostic evaluation demonstrated asymmetry and densification of the subcutaneous tissues of the right mandibular region, associated with fat stranding around the masticator space. Although its initial presentation may suggest an inflammatory origin, several differential diagnoses should be considered.

The main differential diagnoses considered for infiltrative lesions of the masticator space are summarized in Table 1. 

**Table 1:** Differential diagnosis of infiltrative masticator-space lesions

Diagnosis	Typical Features	Distinguishing Findings
Odontogenic infection/abscess	Pain, swelling, fever, dental source	Response to antibiotics; fluid collection on imaging
Chronic osteomyelitis	Pain, trismus, mandibular involvement	Cortical bone destruction, sequestration
Salivary gland malignancy	Parotid mass, facial nerve dysfunction	Primary salivary gland lesion on imaging
Soft tissue sarcoma	Deep infiltrative soft tissue mass	Histopathological confirmation
Metastatic head and neck carcinoma	Cervical adenopathy, mucosal primary tumor	Endoscopic or imaging evidence of primary site
IgG4-related disease	Tumefactive inflammatory lesions	Elevated IgG4 levels and characteristic histology
Diffuse large B-cell lymphoma	Rapid growth, B symptoms, nodal involvement	Histopathological diagnosis with immunophenotyping

**Figure 1:**
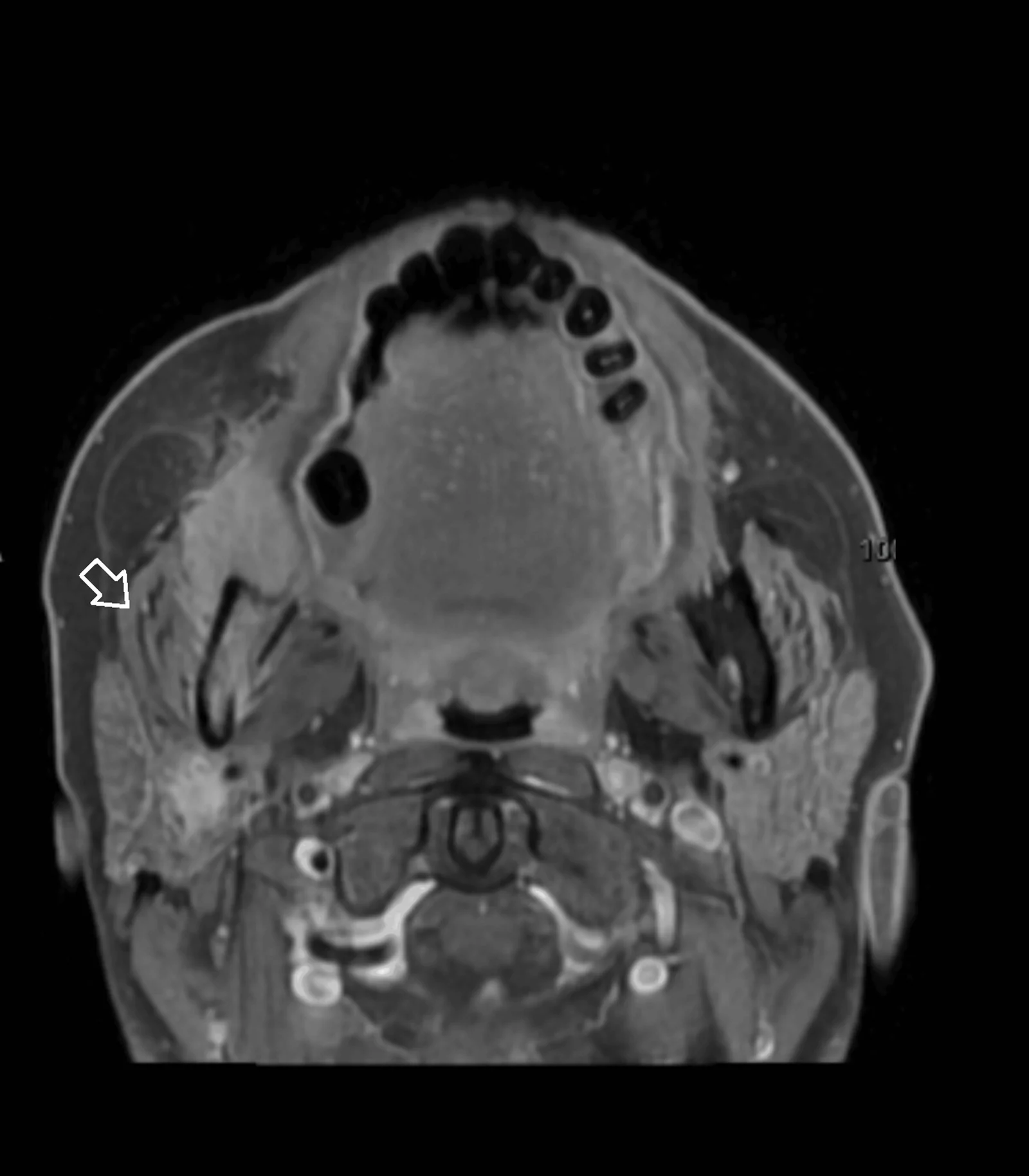
Coronal T2-weighted magnetic resonance imaging (MRI) demonstrating an infiltrative lesion centered in the right masticator space. The white arrow indicates the infiltrative soft tissue lesion extending toward the deep lobe of the right parotid gland.

**Figure 2:**
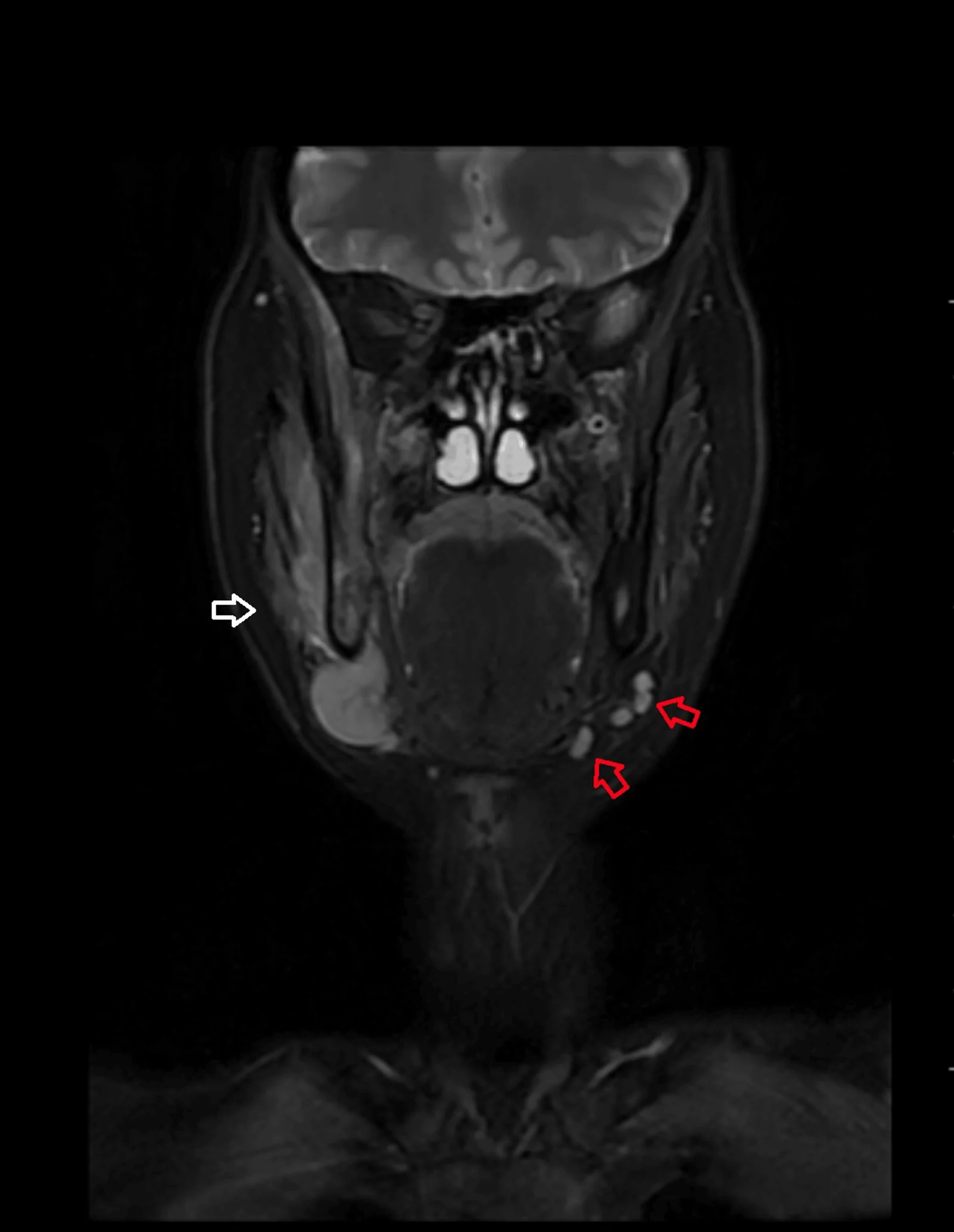
The white arrow indicates the heterogeneous enhancing infiltrative lesion within the right masticator space, while the red arrow indicates ipsilateral cervical lymphadenopathy.

**Figure 3:**
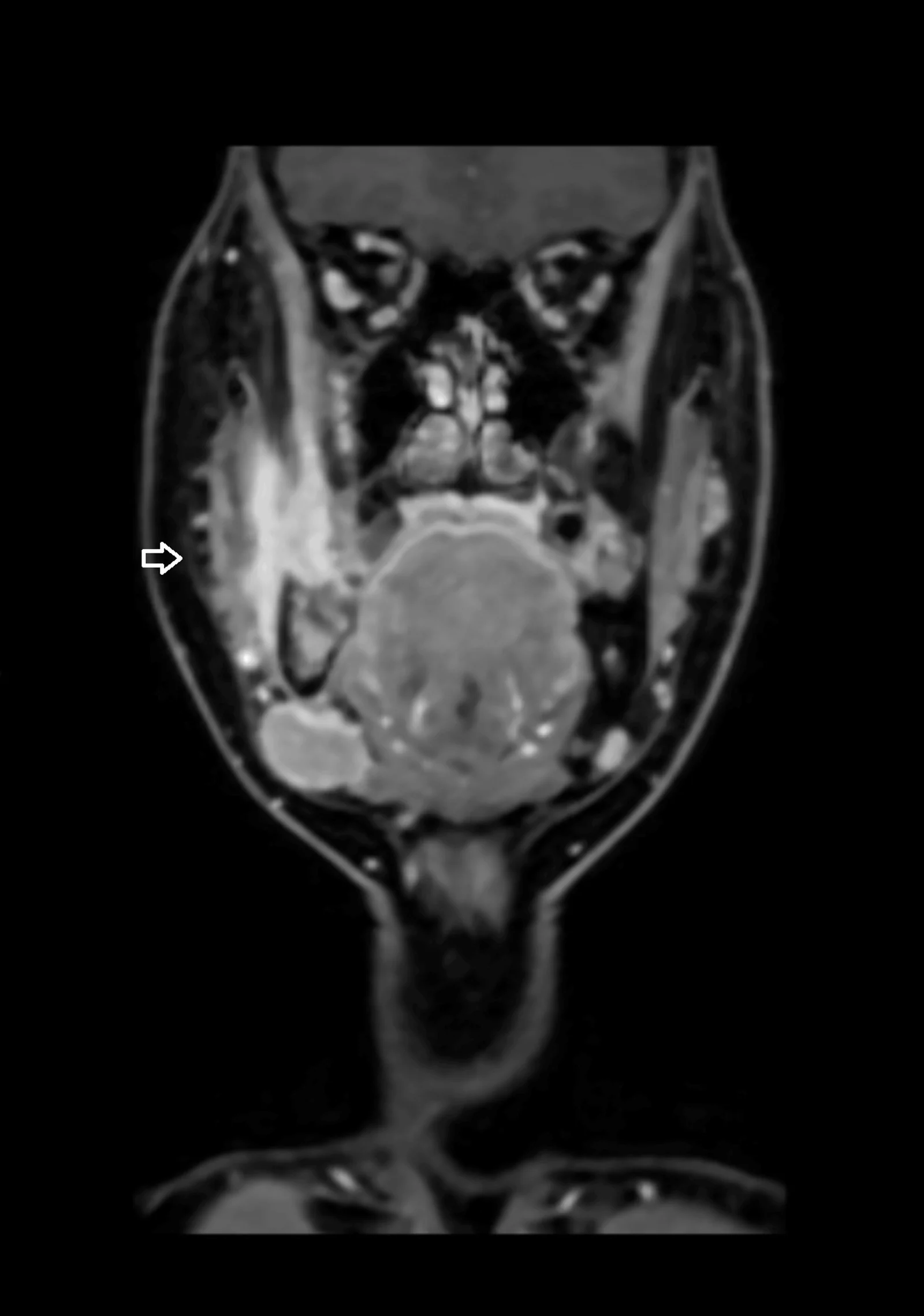
The white arrow indicates infiltration of the right muscles of mastication.

**Figure 4:**
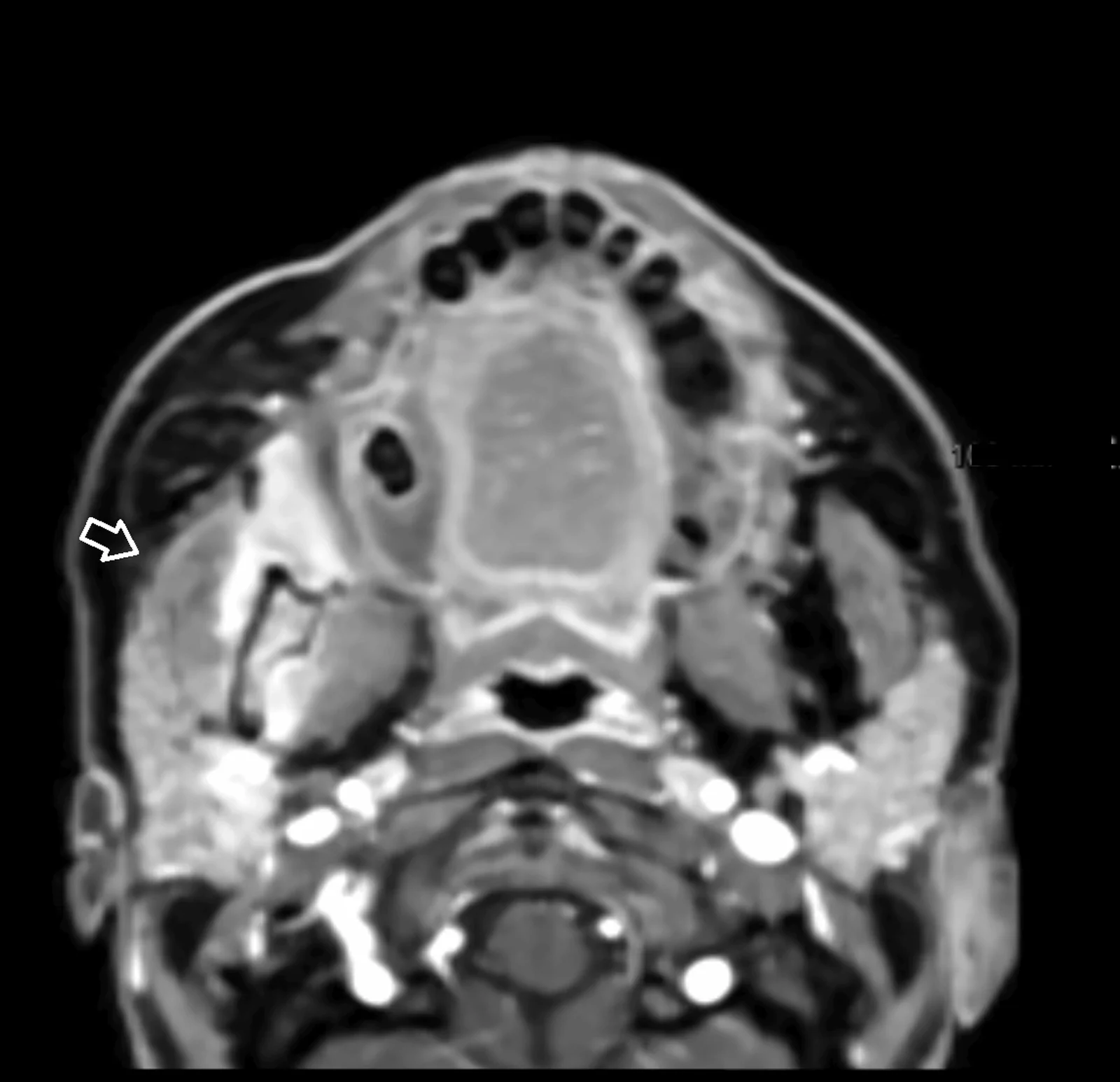
The white arrow indicates the infiltrative lesion extending through the right masticator space.

Magnetic resonance imaging (MRI) performed during further diagnostic evaluation revealed an infiltrative lesion centered within the right masticator space, associated with a necrotic lesion involving the deep lobe of the right parotid gland and ipsilateral cervical lymphadenopathy involving levels IB and II (Figures 1-4).

Fine-needle aspiration (FNA) performed during the diagnostic work-up demonstrated a lymphoid population with numerous blastoid cells and irregular nuclei, raising suspicion for lymphoma but remaining non-diagnostic.

The patient was subsequently referred to Hematology. Physical examination demonstrated swelling of the right mandibular region, trismus, and a right submandibular lymph node measuring approximately 4 cm. Laboratory investigations, including complete blood count and lactate dehydrogenase (LDH) levels, were within normal limits.

A first biopsy performed during the diagnostic work-up was non-diagnostic because of insufficient tissue. Due to persistent clinical suspicion, an excisional cervical lymph node biopsy was subsequently performed.

Positron emission tomography/computed tomography (PET/CT) performed during staging evaluation demonstrated intense fluorodeoxyglucose (FDG) uptake involving the right mandibular region and masticator space, with associated uptake in the right parotid, submandibular, and cervical lymph nodes. No evidence of distant disease was identified.

Histopathological examination of the excisional lymph node biopsy demonstrated DLBCL associated with high-grade follicular lymphoma (transformation). Immunohistochemistry showed CD20+, PAX5+, CD10+, BCL6+, MUM1-, and diffuse CD30 expression, consistent with a germinal center B-cell subtype according to the Hans algorithm. EBER was negative, and MYC and BCL2 protein expression were negative. Molecular studies for MYC, BCL2, and BCL6 rearrangements were not available.

A bone marrow biopsy was performed and showed no evidence of neoplastic infiltration.

According to the Lugano Classification, the disease was classified as Ann Arbor stage IIE [6], with an age-adjusted International Prognostic Index (aaIPI) score of 0 [7], reflecting normal LDH levels, Eastern Cooperative Oncology Group (ECOG) performance status 1, and limited-stage disease.

The patient received four cycles of rituximab, cyclophosphamide, doxorubicin, vincristine, and prednisone (R-CHOP). Post-treatment PET/CT demonstrated a complete metabolic response, with CT showing marked regression of the infiltrative lesion and complete resolution of the cervical lymphadenopathy. Follow-up MRI revealed residual edema of the right mandibular ramus without evidence of active disease. Long-term surveillance imaging showed no evidence of recurrence

Additional surveillance imaging performed during long-term follow-up demonstrated no evidence of recurrent disease within the masticator space or cervical region.

At the most recent hematology follow-up, more than three years after completion of treatment, the patient remained asymptomatic, with ECOG performance status 0, no palpable lymphadenopathy, normal laboratory results, and no evidence of disease recurrence.

The chronological sequence of the patient's diagnostic evaluation, treatment, and follow-up is summarized in Table 2.

**Table 2:** Timeline of the patient's clinical course DLBCL, diffuse large B-cell lymphoma; MRI, magnetic resonance imaging; CT, computed tomography; PET, positron emission tomography; R-CHOP, rituximab, cyclophosphamide, doxorubicin, vincristine, and prednisone.

Clinical course	Clinical event
Approximately five months before the initial presentation	Onset of right mandibular swelling
At the initial presentation	Initial CT scan suggesting an inflammatory process
Approximately two and a half months after the initial presentation	MRI demonstrating an infiltrative lesion involving the right masticator space
At the same diagnostic evaluation	Fine-needle aspiration suspicious for lymphoma
Approximately three weeks later	Initial hematology evaluation
Approximately two weeks later	First biopsy (non-diagnostic)
Approximately one month later	Excisional cervical lymph node biopsy
Approximately one week later	PET/CT staging
Approximately seven weeks later	Histopathological confirmation of DLBCL Ann Arbor stage IIE, with an age-adjusted International Prognostic Index (aaIPI) score of 0
Approximately eight weeks later	Bone marrow biopsy
Within the following week	First cycle of R-CHOP chemotherapy
After four cycles of chemotherapy	Completion of R-CHOP treatment
Approximately three weeks after treatment completion	PET/CT demonstrating complete metabolic response
Approximately four months after treatment completion	CT demonstrating marked regression of the lesion
Approximately seven months after treatment completion	MRI showing no evidence of active lymphoproliferative disease
Approximately two and a half years after treatment completion	CT demonstrating no evidence of recurrent disease
More than three years after treatment completion	Hematology follow-up confirming ongoing complete remission

## Discussion

DLBCL is the most common subtype of non-Hodgkin lymphoma in adults and accounts for approximately one-third of all newly diagnosed cases worldwide [1]. Although extranodal disease is common in DLBCL, primary involvement of the masticator space is rare, often mimicking other head and neck conditions [2-4]. Only a limited number of DLBCL cases involving the masticator space or muscles of mastication have been reported (Table 3) [8-11], including primary masseter involvement, infiltrative masticator-space disease, and lesions extending into adjacent facial compartments. Common presenting features include facial swelling, pain or paresthesia, and nonspecific imaging findings, frequently leading to an initial consideration of inflammatory or odontogenic disease.

**Table 3:** Published cases of DLBCL involving the masticator space or muscles of mastication. DLBCL, diffuse large B-cell lymphoma; R-CHOP, rituximab, cyclophosphamide, doxorubicin, vincristine, and prednisone.

Author, year	Age/Sex	Site/presentation	Histology	Treatment/outcome
Guastafierro et al., 2011 [8]	49/M	Right masseter muscle; three-month history of progressive facial asymmetry, cheek swelling, paresthesia, and burning sensation	Primary DLBCL, stage IEA	Rituximab-based chemotherapy; complete remission with no recurrence during 72-month follow-up
Lim et al., 2023 [9]	70/M	Left masseter muscle; rapidly progressive painless cheek swelling	DLBCL confirmed by ultrasound-guided core needle biopsy	Chemotherapy; favorable clinical response following three treatment cycles
Fan et al., 2024 [10]	56/M	Left masticator space with extension to the premaxillary region and infratemporal fossa; associated cervical, mediastinal, and extranodal disease	DLBCL with aberrant CD5 and CD8 coexpression	R-CHOP chemotherapy; rapid initial tumor regression, followed by fatal Klebsiella sepsis with refractory neutropenia
Arshad et al., 2025 [11]	56/M	Chin/buccogingival mass with an additional lesion involving the left masticator space above the lateral pterygoid muscle; initially treated as odontogenic infection	Germinal center B-cell-like DLBCL	Rituximab-based chemotherapy; marked metabolic response with no evidence of recurrence on follow-up
Present case	34/F	Right masticator space with deep parotid and cervical lymph node involvement	Germinal center B-cell-type DLBCL associated with high-grade follicular lymphoma	Four cycles of R-CHOP; complete metabolic response; ongoing remission >three years

As illustrated in Table 3, previously reported cases share several clinical and radiological features with our patient, highlighting the diagnostic challenge posed by this uncommon presentation.

The masticator space is a deep facial compartment containing the muscles of mastication, mandibular ramus, mandibular division of the trigeminal nerve, and associated vascular structures [4]. Lesions arising in the masticator space commonly present with facial swelling, pain, and trismus, while sensory abnormalities may occur when the mandibular division of the trigeminal nerve is involved [4,5]. Consequently, lymphoma is often not among the first diagnostic considerations, particularly in younger patients.

Our patient initially underwent CT imaging that suggested inflammatory changes within the mandibular region and surrounding soft tissues. Similar nonspecific imaging presentations have been described, with lymphoma appearing as diffuse soft tissue infiltration without the classic appearance of a discrete nodal mass [5]. MRI subsequently provided superior anatomical characterization, demonstrating an infiltrative lesion involving the masticator space, deep parotid lobe, and cervical lymph nodes. Nevertheless, imaging alone remained insufficient to establish a definitive diagnosis.

An important learning point from this case is that obtaining adequate tissue is essential for the diagnosis of lymphoma. In our patient, FNA was insufficient for definitive classification. A subsequent excisional lymph node biopsy provided adequate tissue architecture and allowed complete histopathological and immunophenotypic evaluation, which remains the recommended approach when lymphoma is suspected [1,2]. Histopathological examination demonstrated a germinal center B-cell-type DLBCL according to the Hans algorithm, supported by an immunophenotype of CD20+, CD10+, BCL6+, MUM1−, and diffuse CD30 expression, associated with high-grade follicular lymphoma. Histologic transformation of follicular lymphoma into DLBCL is a well-recognized event associated with a more aggressive clinical course and poorer prognosis [12]. Although the coexistence of DLBCL and high-grade follicular lymphoma may represent histologic transformation, the available pathological findings in our case did not allow definitive distinction between transformation and composite lymphoma. In our case, despite the presence of B symptoms, staging investigations revealed limited-stage disease involving the primary extranodal lesion and ipsilateral cervical lymph nodes, without evidence of distant dissemination. PET/CT was essential for accurate staging, assessment of disease extent, post-treatment response evaluation, and long-term surveillance according to the Lugano response criteria [6].

According to the Lugano Classification, the disease was classified as Ann Arbor stage IIE [6]. The aaIPI score was 0 [7]. Limited-stage DLBCL generally carries an excellent prognosis when treated with modern immunochemotherapy regimens [13].

The patient received four cycles of R-CHOP, consisting of rituximab, cyclophosphamide, doxorubicin, vincristine, and prednisone, achieving complete metabolic remission on post-treatment PET/CT [6]. This regimen is an accepted treatment strategy for selected patients with limited-stage DLBCL and favorable prognostic features. The favorable outcome observed in this case is consistent with the long-term survival benefit demonstrated for R-CHOP-based therapy in patients with DLBCL [14].

This case illustrates that even aggressive lymphomas may remain highly curable when recognized promptly and treated appropriately. Another noteworthy aspect of this case is the persistence of residual facial numbness and mandibular discomfort after treatment completion.

Subsequent imaging demonstrated treatment-related osseous and soft tissue changes without evidence of active lymphoma. Distinguishing residual anatomical abnormalities from recurrent disease represents a common challenge during post-treatment surveillance. Therefore, interpretation of follow-up imaging should integrate anatomical findings with clinical assessment and metabolic response evaluation using PET/CT according to Lugano response criteria [6]

From a primary care perspective, progressive facial swelling associated with trismus, sensory changes, and constitutional symptoms should prompt urgent investigation and specialist referral. Awareness of lymphoma as a potential cause of infiltrative masticator-space lesions may facilitate earlier diagnosis and reduce delays in treatment initiation, particularly when initial imaging findings are inconclusive and symptoms persist despite conservative management.

## Conclusions

DLBCL should be considered in the differential diagnosis of infiltrative lesions involving the masticator space, particularly when accompanied by progressive facial swelling, trismus, sensory abnormalities, cervical lymphadenopathy, or constitutional symptoms. This case highlights the diagnostic limitations of FNA and small tissue samples in suspected lymphoproliferative disorders, emphasizing the importance of obtaining adequate tissue for histopathological characterization.

Long-term remission exceeding three years after treatment further demonstrates the excellent prognosis that can be achieved in limited-stage disease when prompt diagnosis and appropriate immunochemotherapy are achieved. Awareness of atypical extranodal presentations may facilitate earlier diagnosis and timely initiation of potentially curative therapy.
